# Hypothyroïdie et myasthénie: à propos d’un cas

**DOI:** 10.11604/pamj.2019.34.59.10144

**Published:** 2019-09-30

**Authors:** Houda Salhi, Farida Ajdi

**Affiliations:** 1 Service d’Endocrinologie-Diabétologie, CHU Hassan II, Faculté de Médecine et de Pharmacie, Université Sidi Mohamed Ben Abdellah, Fès, Maroc; 2 Endocrinologie et Maladies Métaboliques, Faculté de Médecine et de Pharmacie Université Ibn Zohr, Agadir, Maroc

**Keywords:** Myasthénie, hypo-thyroïdie, thyroïdite de Hashimoto, Myasthenia, hypothyroidism, Hashimoto, thyroiditis

## Abstract

Plusieurs désordres d’origine immunologique spécifiquement thyroïdiens ont été reportés chez les patients myasthéniques. Cette relation reste peu élucidée mais une réaction immunologique croisée entre la jonction neuromusculaire et les composants de la thyroïde a été retrouvé dans la myasthénie et la maladie de Basedow. Il est généralement admis que l’association hyperthyroidie et myasthénie est beaucoup plus fréquente que celle entre myasthénie et hypothyroïdie. Toutefois, aucune explication claire n'a été proposée pour expliquer de cette différence. Nous rapportons le cas d’une patiente âgée de 53 ans qui était suivie au début pour une hypothyroïdie sur probable thyroidite de Hashimoto bien équilibrée sous traitement substitutif et dont l’évolution a été marquée par la découverte d’une myasthénie au cours du suivie. Cette dernière a bien répondu au traitement par néostigmine avec une bonne évolution.

## Abstract (en)

Several disorders of immunological origin, in particular thyroid disorders have been reported in patients with myasthenia. This relationship has been little elucidated but in patients with myasthenia and Graves’ disease was found an immunological cross-reaction between the neuromuscular junction and thyroid components. It is generally accepted that the association between hyperthyroidism and myasthenia is much more common than between myasthenia and hypothyroidism. However, there has been no clear explanation for this difference. We here report the case of a 53-year old patient treated for hypothyroidism due to suspected Hashimoto's disease well balanced under replacement therapy and whose evolution was marked by the detection of myasthenia during the follow up period. Myasthenia responded well to treatment based on neostigmine.

## Introduction

La myasthénie, ou « myasthenia gravis », est une maladie auto-immune rare due à des auto-anticorps spécifiques qui induisent un dysfonctionnement de la transmission neuromusculaire dont la conséquence est une fatigabilité excessive de la musculature striée à l’effort [1]. Elle est plus fréquente chez la femme que chez l’homme. Son diagnostic n’est pas aisé et elle peut rester longtemps méconnue. Elle peut être associée à d’autres affections auto-immunes comme les dysthyroidies en particulier auto-immunes. Les patients myasthéniques peuvent développer des thyropathies d’origine auto immune avant la découverte de la myasthénie, concomitamment ou survenir après. De ce fait, une recherche de désordre thyroïdien d’origine auto immune doit être préconisée au cours de la surveillance des patients myasthéniques. Nous rapportons le cas d’une patiente qui a été suivie pour hypothyroïdie et qui a développée une myasthénie au cours du suivi.

## Patient et observation

Nous rapportons l’observation d’une patiente âgée de 53 ans sans antécédents médicaux notables, et ayant une notion de goitre chez une sœur. La patiente consulte dans notre formation pour asthénie. Examen clinique: absence de signes clinique de dysthyroidie et l’examen cervical: pas de goitre. Un bilan thyroïdien a été demandé devant la notion d’asthénie et les antécédents chez la sœur qui était en faveur d’une hypothyroïdie. Bilan initial: thyréostimuline (TSH): 6μUI/ml (0,35- 5), anticorps anti thyroperoxydase très élevés (élevés (36 x la normale) confirmant l’origine auto-immune. L’échographie thyroïdienne était normale. La patiente a été mise sous traitement substitutif à base de lévothyroxine à la dose de 75ug/jour avec normalisation de sa TSH. Un an plus tard la patiente rapporte l’apparition d’un syndrome myasthénique fait d’une fatigabilité musculaire anormale à l’effort prédominant à la racine des membres). Cette fatigabilité variable dans la journée est plus marquée le soir que le matin et s’atténue au repos. Le bilan a objectivé des anticorps anti récepteurs à l’acétylcholine négative ainsi que le dosage des anticorps anti myosite et anticorps anti-nucléaire. Par ailleurs l’électromyogramme (EMG) a montré la présence d’un bloc neuromusculaire post synaptique aux membres inférieurs. La patiente a été mise néostigmine 3cp par jour avec bonne amélioration.

## Discussion

La myasthénie, ou « myasthenia gravis », est une maladie auto-immune due à des auto-anticorps spécifiques responsables d’un dysfonctionnement de la transmission neuromusculaire induisant une fatigabilité musculaire. Elle peut être associée à de nombreuses maladies, au premier rang desquelles une dysthyroïdie [2]. En 1908, Rennie G décrit pour la première fois l’association entre maladie de Basedow et myasthénie [3]. Depuis lors, cette association a souvent été rapportée par plusieurs auteurs [4, 5]. Bien que le lien entre ces deux maladies auto-immunes restent incertaines; mais une réaction immunologique croisée entre la jonction neuromusculaire et les composants de la thyroïde a été retrouvé dans la myasthénie et la maladie de Basedow [6]. La prévalence des dysthyroïdies auto-immunes chez les myasthéniques est en moyenne supérieure à celle dans la population générale [7]. Ainsi, l’association de ces deux pathologies est plus qu’une coïncidence [7]. En effet, 9% des hommes et 18% des femmes atteintes de myasthénie présentent des troubles thyroïdiens [8, 9]. Ces derniers sont surtout à type d’hyperthyroïdie (17,5%), de goitre simple (1,7%) ou plus rarement d’hypothyroïdie (0,4 à 0,7%) [7]. Par ailleurs, Sahay *et al.* ont rapporté une série de 260 patients myasthéniques, 8 patients seulement parmi eux avaient une thyropathie associée (5 patients avaient une hypothyroïdie et 3 avaient hyperthyroïdie) [10]. Mamarabadi *et al.* ont rapporté une série de 58 patients présentant une myasthénie, 4 patients avaient des troubles thyroïdiens; 3 présentaient une hypothyroïdie et un présente une hyperthyroïdie [3]. L’association entre myasthénie et une hypothyroïdie n’est pas si rare que cela mais sous diagnostiquée du faite de la non spécificité des signes cliniques surtout en cas d’hypothyroïdie infra clinique. Le bilan de notre patiente a objectivé une hypothyroïdie sur une probable thyroïdite de Hashimoto. Notre patiente avait un taux d’anticorps anti thyroperoxydase très élevé à 36 X normale mais l’échographie thyroïdienne n’a pas objectivé de goitre thyroïdien. La thyroïdite de Hashimoto peut se présenter sous différentes formes, les deux principales étant la forme goitreuse et la forme atrophique. La thyroïde peut aussi être de volume normal [11]. Le diagnostic para clinique la myasthénie repose sur le dosage des auto anticorps spécifiques (Anticorps anti-récepteurs de l’acétylcholine, Anticorps anti-MuSK), réalisation d’un électromyogramme et le test thérapeutique aux anti cholinestérasiques. Les anticorps anti récepteurs à l’acétylcholine sont positifs dans 85% des cas de myasthénie généralisée et 66% des cas de myasthénie oculaire [12]. Le dosage des anticorps chez notre patiente était négatif mais électromyogramme a objectivé un bloc neuromusculaire post synaptique aux membres inférieurs et elle a bien répondu au traitement anticholinestérasiques avec une nette amélioration des symptômes.

## Conclusion

L’association myasthénie et dysthyroidies est relativement rare. Une surveillance nous paraît indiquée chez les myasthéniques à la recherche des signes de dysthyroidies et de manifestations myasthéniques chez les patients porteurs d’une dysthyroidie.

## Conflits d’intérêts

Les auteurs ne déclarent aucun conflit d'intérêts.
